# Cardiovascular Risk in Patients with Ankylosing Spondylitis

**DOI:** 10.3390/jcm13206064

**Published:** 2024-10-11

**Authors:** Aleksandra Klisic, Jelena Kotur-Stevuljevic, Osman Cure, Bayram Kizilkaya, Fatma Beyazal Celiker, Huseyin Er, Filiz Mercantepe

**Affiliations:** 1 Faculty of Medicine, University of Montenegro, 81000 Podgorica, Montenegro; aleksandranklisic@gmail.com; 2 Center for Laboratory Diagnostics, Primary Health Care Center, 81000 Podgorica, Montenegro; 3 Department for Medical Biochemistry, Faculty of Pharmacy, University of Belgrade, 11221 Belgrade, Serbia; jkotur@pharmacy.bg.ac.rs; 4 Department of Rheumatology, Faculty of Medicine, Recep Tayyip Erdogan University, Rize 53100, Turkey; 5 Department of Internal Medicine, Faculty of Medicine, Recep Tayyip Erdogan University, Rize 53100, Turkey; bayram.kizilkaya@saglik.gov.tr; 6 Department of Radiology, Faculty of Medicine, Recep Tayyip Erdogan University, Rize 53100, Turkey; fatma.bceliker@erdogan.edu.tr (F.B.C.); huseyin.er@erdogan.edu.tr (H.E.); 7 Department of Endocrinology and Metabolism, Faculty of Medicine, Recep Tayyip Erdogan University, Rize 53100, Turkey

**Keywords:** ankylosing spondylitis, BASDAI score, cardiometabolic risk factors, cIMT, principal component analysis

## Abstract

**Objectives:** Ankylosing spondylitis (AS) is an autoinflammatory, chronic disease. Patients with AS are at increased risk of cardiovascular disease (CVD). The link between AS and subclinical atherosclerosis is multifactorial and still not completely understood. The aim of this study was to examine the potential associations between carotid intima–media thickness (cIMT) and different cardiometabolic biomarkers in individuals with AS. **Methods:** A total of 96 patients with AS were prospectively included. cIMT was measured via ultrasonography. Multiple linear regression analysis was used to find the best predictors of cIMT values. Principal component analysis (PCA) was implemented to extract factors that were further tested via binary logistic regression analysis in relation to cIMT. **Results:** Waist circumference (WC), low-density lipoprotein cholesterol (LDL-c), and the BASDAI score were independently correlated with cIMT in AS patients (*p* = 0.037, *p* = 0.060, and *p* = 0.048, respectively; adjusted R^2^ = 0.113). PCA extracted four panels of biomarkers, i.e., “haematology–lipid-related factor” (i.e., ferritin, haemoglobin, HDL-c, and triglycerides), “proinflammatory–prothrombotic-related factor” (i.e., platelets, neutrophils, and C-reactive protein), “LDL-c–vitamin-related factor” (i.e., vitamins D and B12, and LDL-c), and “age–glucometabolic-related factor” (i.e., age and HbA1c), in relation to higher cIMT in patients with AS. Among these four clusters, “age–glucometabolic-related factor” was an independent predictor of increased cIMT (*p* < 0.001). **Conclusions:** In addition to traditional cardiometabolic risk factors, WC and LDL-c, the disease activity score (BASDAI) is independently related to subclinical atherosclerosis in AS patients. The joint involvement of heterogeneous cardiometabolic risk factors may reflect different pathophysiological processes of subclinical atherosclerosis in patients with AS.

## 1. Introduction

Ankylosing spondylitis (AS) is an autoinflammatory, chronic disease that affects not only the sacroiliac joints and spine but also the cardiovascular system, skin, kidneys, gastrointestinal tract, lungs, and eyes [1,2]. AS has a long asymptomatic stage, and it is assumed that genetic factors and the activated inflammatory cascade are the pathophysiological mechanisms of the disease [2]. Given the underlying chronic inflammation in AS, these individuals are at increased risk of CVD [3]. Compared with the general population, subjects with AS are at a 30–50% risk of incident cardiovascular events [4].

Endothelial dysfunction and subclinical atherosclerosis can be observed in patients with AS even without manifesting CV risk factors [2]. Endothelial dysfunction is the first and reversible stage of atherosclerosis onset [5]. Atherosclerosis, the principal cause of CVD, involves an enhanced immune-inflammatory response, a disbalance between antioxidants and pro-oxidants, lipid peroxidation, endothelial injury and dysfunction, compromised vasodilatation, perturbed haemostasis, and an enhanced prothrombotic state. All such disturbances promote the development of atherosclerotic plaques that, if ruptured, precede CVD [5]. However, the link between AS and subclinical atherosclerosis is multifactorial and still not completely understood [1].

The identification of individuals with high CVD risk is of paramount importance, and such patients would benefit from an immediate therapeutic strategy before experiencing CVD onset and its complications. Although much progress has been made in identifying novel endothelial dysfunction biomarkers that could be reliable parameters of subclinical atherosclerosis [1,5,6,7], traditional biomarkers should not be neglected, especially concerning their joint involvement in different pathophysiological processes of subclinical atherosclerosis.

Carotid intima-media thickness (cIMT) reflects the degree of endothelial dysfunction and is regarded as a simple, cost-effective, non-invasive surrogate parameter of subclinical atherosclerosis [8]. Indeed, previous studies, although with relatively small sample sizes, reported higher cIMT in patients with AS than in healthy individuals [2,3,9,10]. To gain deeper insight into the pathophysiological traits of AS and subclinical atherosclerosis, the aim of the current study was to examine the potential associations between cIMT and different cardiometabolic biomarkers, such as proinflammatory, prothrombotic, and proatherogenic biomarkers, in individuals with AS via a thorough statistical approach, such as principal component analysis.

## 2. Materials and Methods

### 2.1. Study Population

A total of 96 patients diagnosed with AS according to the Assessment in Spondylarthritis International Society (ASAS) criteria [11] were prospectively included in this study involving a consultation-based cohort. The participants’ age, sex, smoking status, alcohol consumption status, medication use, family disease history, and disease duration were recorded. Those who smoked, consumed alcohol, had active and chronic infections, dyslipidaemia, diabetes mellitus, hypertension, coronary artery disease, or malignant diseases, had any spine surgeries, used steroids, lipid-lowering agents, or antihyperglycemic agents, were pregnant, or were lactating were excluded from this study. This study was conducted in accordance with the Declaration of Helsinki (ethical approval no: E-40465587-050.01.04-1041, 2024/92). Informed consent was obtained from all participants.

### 2.2. Measurement of Demographic, Clinical, Disease Activity, and Laboratory Parameters

The participants’ physical examination was performed, and height and weight measurements were taken. Body mass index (BMI) was calculated by dividing weight (kilograms) by height (meters) squared. The BATH Ankylosing Spondylitis Disease Activity Index was measured in the patients [12].

Blood samples were taken after at least eight hours of fasting. The complete blood count (including white blood cell (WBC) count, neutrophils, haemoglobin, and platelets), C-reactive protein levels (CRP), and lipid levels, including triglycerides, total cholesterol (TC), high-density lipoprotein cholesterol (HDL-c), low-density lipoprotein cholesterol (LDL-c), folate, ferritin, vitamin B12, and vitamin D, were analysed.

### 2.3. Carotid Artery Intima–Media Thickness Measurement

Carotid IMT was measured by two experienced radiologists using the LA2-14A probe on the Samsung RS85 Prestige device. In images obtained in the sagittal plane during greyscale ultrasonography, the intima-media layer was measured at the 1 cm proximal level of the carotid bifurcation distal to the common carotid artery, with appropriate magnification. If there was an atherosclerotic plaque on the walls of the common internal carotid artery at all levels within the examination area, it was noted.

### 2.4. Statistical Analysis

The results are presented as the median and 25th to 75th percentile values because of a non-normal distribution. Differences between groups were examined by the Kruskal–Wallis and Mann–Whitney U tests. Bivariate correlations were analysed via Spearman’s correlation analysis. Multiple linear regression (MLR) analysis with backwards selection was used to find the best predictors of cIMT values. Factorial analysis (principal component analysis, PCA) with varimax-normalized rotation was implemented to extract factors that were further tested via binary logistic regression analysis. In particular, an eigenvalue >1 was used to extract the factors, whereas variables with factor loadings >0.5 were included in further PCA. Analysis adequacy was tested via the Kaplan–Meier–Olkin (KMO) test and Bartlett’s sphericity test. Binary logistic regression analysis was used to test a possible independent association of PCA-produced factors (scores) with high cIMT values. Statistical analysis was performed via PASW^®^ Statistic v.18 (Chicago, IL, USA) software, and *p* values <0.05 were considered statistically significant.

## 3. Results

Table 1 shows the demographic, general clinical, and biochemical characteristics of the AS patients included in this study.

This group of patients was considered younger (the median age was less than 40 years), with more men than women (60% vs. 40%). The patients were not generally alcohol users (approximately 6%), and 38.5% of them were smokers. The dominant group of patients (almost 40%) had a university degree, and more than 70% were married. The delay in diagnosis for approximately 75% of the patients was less than 3 months. Approximately 50% of the patients had a positive family history of AS. The patients did not have any comorbidities other than inflammatory back pain (more than 93%), whereas 16% of the patients had peripheral arthritis or uveitis. Enthesitis occurred in 26% of the patients. The majority of patients (79%) had bilateral enhancement of the disease documented by MRI. With respect to AS therapy, 34% of the patients did not use any drugs, whereas 29% of the patients used two different drugs. The average disease duration was 5 years.

Table 2 shows the biochemical and clinical parameter comparisons in the cIMT tertile subgroups of ankylosing spondylitis patients. Older patients and patients with higher BMIs, WCs, WtHRs, and total cholesterol concentrations, such as LDL-c and HbA1c, had significantly higher cIMT values in the current study. The folate concentration was the highest in the patients with the highest cIMT values. Additionally, the BASDAI score was significantly greater in the third cIMT tertile group.

cIMT was significantly positively correlated with age, BMI, WC, WtHR, total and LDL-c concentrations, and HbA1c level (Table 3). These correlations are expected because all of them are well-established risk factors for atherosclerosis, and cIMT is a physical measure of plaque magnitude.

Considering the existing relationship between cIMT levels and other patient-related parameters which are characteristic of the disease, we performed MLR to find the best model of cIMT value predictors. This analysis is presented in Table 4. Parameters were selected among those that, in Spearman’s nonparametric correlations, showed a significant correlation with cIMT. The starting model consisted of the following parameters: BASDAI score, LDL-c, triglycerides, BMI, folate, and WC. This analysis enabled the best model selection, which comprises the parameters WC, LDL-C, and BASDAI score, with an adjusted R^2^ = 0.113.

Factorial analysis (principal component analysis, PCA) was implemented to reduce the number of variables to a small number of factors grouped by the same variability. Additionally, this analysis enabled the factors’ transformation into numerically characterized variables, i.e., scores, which could be further used for subsequent analysis to test their predictive ability. The total model explained 41% of the variability in the set of parameters. Analysis adequacy was confirmed by the KMO measure of sample adequacy (0.551) and Bartlett’s test of sphericity (*p* < 0.001). The detailed results are presented in Table 5.

The first factor, the haematology- and lipid-related factor, included ferritin, haemoglobin, HDL-c, and triglycerides, among which only HDL-c had negative loading, and this factor explained 12% of the data variability. The second factor (proinflammatory–prothrombotic-related factor) consisted of three parameters, platelet count, neutrophil count, and CRP, all of which had positive loadings, explaining 11% of the variability. The third factor was the LDL-c–vitamin-related factor, which included vitamins D and B12, as well as the LDL-c concentration, and explained 9% of the total model variation. The fourth factor (age–glucometabolic-related factor) consisted of age and HbA1c, both of which had positive loadings and 9% variability. Afterwards, we performed binary logistic regression analysis with the scores produced in the PCA to test its ability to predict high cIMT (third tertile vs. first tertile of the average cIMT value). The results of this analysis are presented in Table 6.

High cIMT (third tertile and above) was significantly predictive of age-related factors (age and HbA1c).

**Figure 1:**
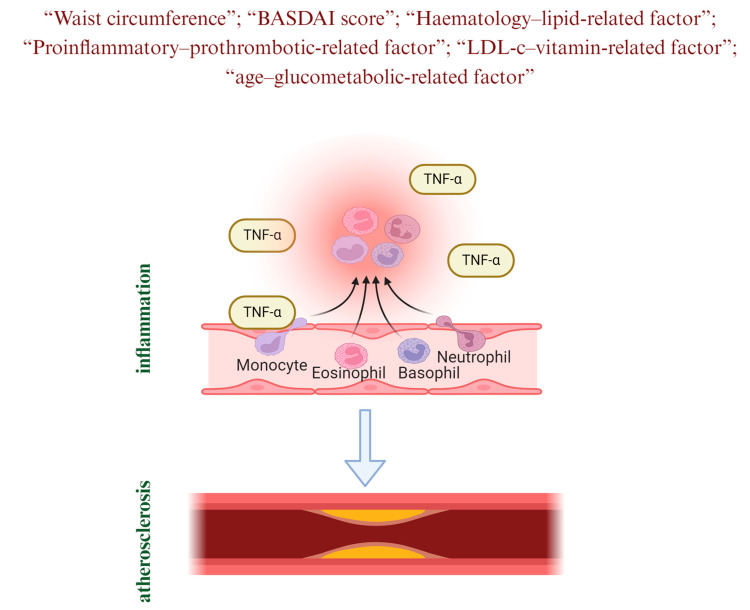
Pathophysiology of cardiovascular disease in ankylosing spondylitis.

A schematic representation created on the basis of the results of the current study is presented in Figure 1.

## 4. Discussion

The current study examined the relationship between AS and subclinical atherosclerosis via heterogeneous parameters, such as biochemical markers and cIMT, as simple, cost-effective, and non-invasive diagnostic tools. To the best of our knowledge, this is the first study that has examined a variety of cardiometabolic factors in relation to cIMT in patients with AS via a comprehensive statistical approach by clustering different cardiometabolic risk factors in relation to subclinical atherosclerosis. Although some of the studies reported no difference in cIMT between AS patients and controls [13,14,15], others reported higher cIMT in AS patients than in healthy individuals [2,3,10,15].

The main findings of the current study indicate that WC, LDL-c, and the disease activity score, i.e., the BASDAI score, are independently correlated with cIMT in AS patients. This is the first study that has reported an independent association between WC and cIMT in patients with AS; the findings were similar between non-AS patients and nondiabetic patients in studies with a cross-sectional [16,17] and longitudinal design [18]. Central obesity was also shown to be significantly associated with cIMT in clinically healthy individuals [18].

Visceral compartments of adipose tissue are a significant source of proinflammatory cytokines that favour an increase in neutrophil infiltration and changes in the functional characteristics of macrophages. The potential of neutrophils to secrete proinflammatory cytokines, reactive oxygen species, and a variety of proteolytic enzymes, along with their ability to invade the vascular wall, can provoke damage to the endothelium, which precedes the development of atherosclerosis [5,19].

The novel finding is also the independent positive correlation between LDL-c and cIMT in AS patients. This finding contrasts with previous findings [8,20,21] that included a smaller sample size than our current study did. In the general population, the results are also contradictory [18,22].

LDL is involved in the early stage of atherosclerosis. The recruitment of inflammatory cells to the subendothelial layer and the promotion of prothrombotic alterations on the cell surface of the endothelium are important properties of LDL. In the presence of ROS (macrophages and smooth muscle cells are the major sources of ROS), LDL becomes oxidized and is thus transformed into oxidized (ox)-LDL, which exerts prothrombotic effects through the activation of platelets and thrombus formation [23].

Our findings of the association between the BASDAI score and cIMT support the hypothesis that disease activity may influence endothelial dysfunction [21]. The obtained results are in line with those of some studies that reported a relationship between the BASDAI score and cIMT [3,21] but oppose those of other studies that reported no relationship between the disease activity score and subclinical atherosclerosis [8,20]. However, Yilmaz et al. [3] reported only a weak but not independent correlation between cIMT and the BASDAI score. Similarly, Verma et al. [21] did not confirm the independent correlation between the BASDAI score and cIMT in AS patients.

There are no previous studies on factorial analysis (PCA) in relation to cIMT in AS patients. In the present study, a factorial analysis extracted four panels of biomarkers, i.e., “haematology- and lipid-related factors” (i.e., ferritin, haemoglobin, HDL-c, and triglycerides), “proinflammatory–prothrombotic-related factors” (i.e., platelet count, neutrophil count, and CRP), “LDL-c–vitamin-related factors” (i.e., vitamins D and B12, and LDL-c), and “age–glucometabolic-related factors” (i.e., age and HbA1c).

Among these four clusters, the latter (i.e., age and HbA1c) showed the ability to independently predict high cIMT (third tertile vs. first tertile of the average cIMT value). The independent relationship between age and cIMT in AS patients has recently been confirmed [3,9]. Herein, we have shown the joint influence of age and HbA1c on increased cIMT in AS patients. Zhou et al. [24] reported that in a study of a Chinese nondiabetic population that included nearly 3500 participants, HbA1c was associated with an increased risk of cIMT. Age exhibited an interaction influence on this relationship, which was more evident in individuals aged <60 years in particular.

Atherosclerosis is a multifactorial process. Increased inflammation and oxidative stress, dyslipidaemia, and a prothrombotic state are related to atherosclerosis [5].

There is an assumption that inflammation per se can worsen the lipid profile in terms of increasing serum LDL-c and triglyceride levels and lowering serum HDL-c and can thus aggravate atherosclerosis, which might at least partially explain the increased cardiovascular risk in patients with AS (1).

To the best of our knowledge, the relationship between ferritin and cIMT has not been previously shown in individuals with AS, whereas such a correlation was previously confirmed in a healthy population [25]. As an acute-phase reactant, serum ferritin levels increase in parallel with the increase in inflammation levels [26], which might in part explain the higher levels of ferritin and dyslipidaemia in the highest tertile of the cIMT group.

Inflammation, oxidative stress, and thrombosis are mutually involved in atherosclerosis progression [7]. In parallel with the enhanced proinflammatory milieu, the process of thrombosis is accelerated. Cytokines may influence the physiological functions, size, and morphology of haematological parameters and aggravate the prothrombotic milieu [27].

In the present study, we found that clusters of higher vitamin D and vitamin B12 and lower LDL-c were related to higher cIMT in AS patients (i.e., “LDL-c–vitamin-related factor”). These unexpected results might be explained by the assumption that some of the patients might have used vitamin D and B12 supplementation, which might reduce cIMT and inflammation [28,29].

One of the strengths of the current study is the relatively larger sample size included compared to that of previous studies [2,3,8,9,10] that examined the association between cIMT and AS. Moreover, we have applied PCA to investigate the relationships between different clusters of cardiometabolic biomarkers and cIMT in patients with AS in greater detail since different panels of biomarkers can reflect different signalling pathways of the disease. One of the limitations of this study is its cross-sectional design, which does not allow us to examine the causality between the examined variables. The inclusion of a control group with propensity score-based matching would be highly important for future investigations to gain deeper knowledge regarding AS and cardiometabolic risk. Additionally, we were limited in terms of data on nutritional habits and vitamin D and B12 supplementation, which might influence the related results. Since this was a single-centre study, multiethnic and multicentre studies are needed to further examine such issues. We were not able to measure lipoprotein subclasses or ox-LDL, which might significantly contribute to a deeper understanding of atherogenic dyslipidaemia in AS.

## 5. Conclusions

In addition to traditional cardiometabolic risk factors such as WC and LDL-c, the disease activity score (BASDAI) is independently related to subclinical atherosclerosis in AS patients. The joint involvement of heterogeneous cardiometabolic risk factors, such as a panel of biomarkers, i.e., “haematology–lipid-related factors” (i.e., ferritin, haemoglobin, HDL-c, and triglycerides), “proinflammatory–prothrombotic-related factors” (i.e., platelet count, neutrophil count, and CRP), “LDL-c–vitamin-related factors” (i.e., vitamins D and B12, and LDL-c), and “age–glucometabolic-related factors” (i.e., age and HbA1c), may reflect different pathophysiological processes of subclinical atherosclerosis in patients with AS. New longitudinal studies are needed to confirm our results.

## Figures and Tables

**Table 1:** General clinical and biochemical data of ankylosing spondylitis patients.

Parameter	AS Patients (N = 96)
Age, years	36.5 (31.0–47.0)
Gender	
Female	38 (39.6)
Male	58 (60.4)
Alcohol use	
NO	90 (93.8)
YES	6 (6.3)
Smoking	
NO	59 (61.5)
YES	37 (38.5)
Educational background	
Primary school	15 (15.6)
Secondary school	14 (14.6)
High school	29 (30.2)
University	38 (39.6)
Marital status	
Single	23 (24.0)
Married	69 (71.9)
Divorced	4 (4.2)
Delay in diagnosis, months	2 (1–3)
Family history of AS	
NO	47 (49.0)
YES	49 (51.0)
Comorbidities	
Diabetes mellitus	
NO	96 (100)
CAD	
NO	96 (100)
Hypertension	
NO	96 (100)
Hyperlipidaemia	
NO	96 (100)
Inflammatory back pain	
NO	6 (6.3)
YES	90 (93.7)
Peripheral arthritis	
NO	81 (84.4)
YES	15 (15.6)
Uveitis	
NO	81 (84.4)
YES	15 (15.6)
Dactylitis	
NO	96 (100)
Enthesitis	
NO	71 (74.0)
YES	25 (26.0)
Sacroiliac MRI enhancement status	
None	6 (6.3)
Right unilateral	7 (7.3)
Left unilateral	7 (7.3)
Bilateral	76 (79.2)
Drugs used to treat AS	
NSAID	33 (34.4)
Anti TNF	
etanercept	12 (12.5)
adalimumab	28 (29.2)
certolizumab	7 (7.3)
infliximab	6 (6.3)
golimumab	10 (10.4)
Drug use duration, years	3 (2–5)
Duration of disease, years	5.0 (4.0–10.0)
BMI, kg/m^2^	25.9 (22.6–29.4)
WC, cm	91.0 (86.0–104.0)
WtHR	0.546 (0.505–0.596)
WBC	7.52 (6.49–8.71)
Neutrophil count, ×10^9^ /L	4.33 (3.34–5.32)
Lymphocyte count, ×10^9^/L	2.39 (1.98–2.92)
Haemoglobin, g/L	140 (120–151)
Platelet count, ×10^9^/L	285 (252–337)
Total cholesterol, mg/dL	209.5 (180.0–237.0)
Triglycerides, mg/dL	109.5 (74.0–173.0)
HDL-c, mg/dL	49.5 (43.0–60.0)
LDL-c, mg/dL	133.5 (108.0–152.0)
Vitamin B12, pg/mL	326.0 (264.0–411.0)
Folate, ng/mL	8.0 (6.9–11.0)
Vitamin D, ng/mL	10.00 (6.00–16.00)
Ferritin, ng/mL	62.5 (22.0–95.0)
Hba1c, %	5.55 (5.20–5.80)
BASDAI score	4.40 (3.40–5.90)

*Abbreviations:* CAD, coronary artery disease; MRI, magnetic resonance imaging; AS, ankylosing spondylitis; NSAID, nonsteroidal anti-inflammatory drug; TNF, tumour necrosis factor; BMI, body mass index; WC, waist circumference; WtHR, waist-to-height ratio; WBC, white blood cell; HDL-c, high-density lipoprotein cholesterol; LDL-c, low-density lipoprotein cholesterol; HbA1c, glycated haemoglobin A1c; BASDAI, bath ankylosing spondylitis disease activity index.

**Table 2:** Comparison of biochemical and clinical parameters among cIMT tertile subgroups of ankylosing spondylitis patients.

	cIMT (mm)			*p*
	≤0.480	0.481–0.565	≥0.566	
Age, years	30 (26–34)	37 (33–42) **	49 (41–52) ***^,###^	<0.001
BMI, kg/m^2^	23.5 (21.4–26.7)	26.8 (23.2–28.7) *	28.5 (24.3–30.9) **	0.007
WC, cm	89.0 (83.5–92.5)	92.0 (86.0–108.0) *	101.0 (88.0–108.0) **	0.011
WtHR	0.511 (0.494–0.534)	0.547 (0.509–0.606) **	0.582 (0.548–0.632) ***	<0.001
Total cholesterol, mg/dL	193.0 (164.5–221.0)	206.0 (177.0–231.0)	224.0 (191.5–241.5) *	0.038
LDL-c, mg/dL	122 (98–150)	127 (110–143)	145 (124–156) *	0.109
HbA1c, %	5.30 (5.10–5.55)	5.50 (5.00–5.80)	5.80 (5.55–6.00) ***^,##^	<0.001
Folate, ng/mL	8.0 (6.0–12.0)	7.9 (5.0–9.0)	9.0 (8.0–11.0) ^##^	0.037
BASDAI score	3.90 (3.25–5.1)	4.00 (3.30–4.90)	5.50 (4.40–6.60) *^,#^	0.043
Right carotid thickness, mm	0.440 (0.400–0.455)	0.520 (0.500–0.560) ***	0.650 (0.620–0.710) ***^,###^	<0.001
Left carotid thickness, mm	0.440 (0.400–0.450)	0.520 (0.500–0.540) ***	0.650 (0.620–0.710) ***^,###^	<0.001

For the P-Kruskal–Wallis test, subgroup comparisons were performed via the Mann–Whitney U test. *, **, and *** *p* < 0.05, 0.01, and 0.001 vs. first cIMT tertile subgroup, respectively. ^#, ##^, and ^###^ *p* < 0.05, 0.01, and 0.001 vs. second cIMT tertile subgroup, respectively. *Abbreviations:* BMI, body mass index; WC, waist circumference; WtHR, waist-to-height ratio; LDL-c, low-density lipoprotein cholesterol; HbA1c, glycosylated haemoglobin A1c; BASDAI, bath ankylosing spondylitis disease activity index.

**Table 3:** Spearman’s nonparametric correlation between the cIMT average value and other parameters.

	cIMT Average
Age, years	0.414 ***
BMI, kg/m^2^	0.213 *
WC, cm	0.253 *
Total cholesterol, mg/dL	0.231 *
LDL-c, mg/dL	0.232 *
HbA1c, %	0.254 *
WtHR	0.328 ***

*Abbreviations:* BMI, body mass index; WC, waist circumference; LDL-c, low-density lipoprotein cholesterol; HbA1c, glycated haemoglobin A1c; WtHR, waist-to-height ratio, * *p* < 0.05; *** *p* < 0.01.

**Table 4:** Multiple linear regression analysis (backwards selection) of the best predictor model.

Parameters (Model’s Adjusted R^2^ = 0.113)	Unstandardized Coefficients		*p*
	B (95th CI)	Std. Error	
WC, cm	0.002 (0.000–0.005)	0.001	0.037
LDL-c, mg/dL	0.001 (0.000–0.001)	0.000	0.060
Bath ankylosing spondylitis disease activity index (BASDAI) score	0.013 (0.000–0.026)	0.006	0.048

CI—confidence interval. *Abbreviations:* WC, waist circumference; LDL-c, low-density lipoprotein cholesterol.

**Table 5:** PCA-derived factors in AS patients.

Factors	Variables Included in the Factor	Loadings of the Variables	Factor Variability, % (Total Variance: 41%)
Haematology–lipid-related factor	Ferritin	0.719	12
	Haemoglobin	0.704	
	HDL-c	−0.536	
	Triglycerides	0.530	
Proinflammatory–prothrombotic-related factor	Platelet count	0.730	11
	Neutrophil count	0.696	
	C-reactive protein	0.661	
LDL-c–vitamin-related factor	Vitamin D level	0.731	9
	Vitamin B12 level	0.621	
	LDL-c	−0.506	
Age–glucometabolic-related factor	Age	0.739	9
	HbA1c	0.707	

*Abbreviations:* HDL-c, high-density lipoprotein cholesterol; LDL-c, low-density lipoprotein cholesterol; HbA1c, glycosylated haemoglobin A1c.

**Table 6:** Univariate binary logistic regression analysis for high cIMT and high BASDAI value prediction.

Factors (Univariate Analysis)	B (SE)	Wald Coeff.	OR (95% CI)	*p*
cIMT				
Age–glucometabolic-related factor	2.084 (0.500)	17.359	8.035 (3.015–21.414)	<0.001

SE, standard error; OR, odds ratio (95th CI, confidence interval); *p*, binary logistic regression analysis. *Abbreviations:* cIMT, carotid intima–media thickness.

## Data Availability

All of the data generated or analysed during this study are included in this article. The data will be available upon reasonable request (contact persons: filiz.mercantepe@saglik.gov.tr and aleksandranklisic@gmail.com).
